# Construction and validation of a metabolic risk model predicting prognosis of colon cancer

**DOI:** 10.1038/s41598-021-86286-z

**Published:** 2021-03-25

**Authors:** Didi Zuo, Chao Li, Tao Liu, Meng Yue, Jiantao Zhang, Guang Ning

**Affiliations:** 1 Department of Endocrinology and Metabolism, The First Hospital of Jilin University, Changchun, Jilin Province China; 2 Department of Colorectal and Anal Surgery, The First Hospital of Jilin University, Changchun, Jilin China; 3 Key Laboratory for Endocrine and Metabolic Diseases of Ministry of Health of China, Shanghai National Clinical Research Center for Endocrine and Metabolic Diseases, Shanghai Institute for Endocrine and Metabolic Diseases, Ruijin Hospital, Shanghai Jiaotong University School of Medicine, Shanghai, China

**Keywords:** Cancer, Cancer genomics, Cancer

## Abstract

Metabolic genes have played a significant role in tumor development and prognosis. In this study, we constructed a metabolic risk model to predict the prognosis of colon cancer based on The Cancer Genome Atlas (TCGA) and validated the model by Gene Expression Omnibus (GEO). We extracted 753 metabolic genes and identified 139 differentially expressed genes (DEGs) from TCGA database. Then we conducted univariate cox regression analysis and Least Absolute Shrinkage and Selection Operator Cox regression analysis to identify prognosis-related genes and construct the metabolic risk model. An eleven-gene prognostic model was constructed after 1000 resamples. The gene signature has been proved to have an excellent ability to predict prognosis by Kaplan–Meier analysis, time-dependent receiver operating characteristic, risk score, univariate and multivariate cox regression analysis based on TCGA. Then we validated the model by Kaplan–Meier analysis and risk score based on GEO database. Finally, we performed a weighted gene co-expression network analysis and protein–protein interaction network on DEGs, and Kyoto Encyclopedia of Genes and Genomes pathways and Gene Ontology enrichment analyses were conducted. The results of functional analyses showed that most significantly enriched pathways focused on metabolism, especially glucose and lipid metabolism pathways.

## Introduction

Colon cancer is a common malignant tumor which mainly occurs in the proximal colon. Colon cancer and rectal cancer are often grouped as “colorectal cancer” (CRC), which is the fourth most common cancer and the second leading cause of cancer-related death in America^1^. There are estimated to be 147,950 newly diagnosed CRC individuals, including 104,610 colon cancer patients in America in 2020^2^. In addition, the colon cancer patients are rapidly shifting younger as a result of older individuals declining and younger individuals increasing^3^. However, colon cancer is potentially preventable because most of the cases and deaths are attributable to an unhealthy lifestyle, including high-fat and low-fiber diet, smoking and drinking, insufficient physical activity, and overweight^4^. Although morbidity and mortality can be mitigated through appropriate screening, including imaging techniques and colonoscopy^5,6^, approximately 80% of CRC patients show recurrence during the first 3 years. Thus, identifying reliable prognostic biomarkers to select high-risk colon cancer patients is important for improving the survival rate.

Recently, metabolic reprogramming has become a hot topic^7^. Studies had been shown that tumor metabolism played a vital role in tumor cells^8,9^. Metabolism plays a vital role in the progression and prognosis of colon cancer^10^. Energy metabolism is the basis of cell proliferation, and change of cell metabolism is the feature of tumor cells^9,11^. Tumor cells change metabolic processes to satisfy the increased energy and nutritional demands, for growth and invasion^12^. The pathogenesis, progression and prognosis of colon cancer is closely related to metabolic progress, including glucose metabolism, amino acid metabolism, and lipid metabolism^13–15^. In this study, we constructed and validated a metabolic risk model of colon cancer based on the data downloaded from The Cancer Genome Atlas (TCGA) and Gene Expression Omnibus (GEO) database, to explore the potential role of metabolic genes and accurately predict prognosis of colon cancer.

## Materials and methods

### Data collection

We retrieved and downloaded gene expression profile and clinical data (age, gender, tumor grade, TMN stage) from TCGA (https://portal.gdc.cancer.gov/). We then obtained and combined the gene expression matrix of the GSE17536, GSE17537, and GSE29621 series from the GEO (http://www.ncbi.nlm.nih.gov/geo/) database. The data was extracted, annotated, and normalized by Strawberry Perl (version5.30.1.1). We downloaded the metabolic genes from Gene Set Enrichment Analysis (GSEA) platform (http://software.broadinstitute.org/gsea/downloads).

### Differentially expressed metabolic genes identification

Metabolic genes were defined as genes enriched in metabolism pathways based on Kyoto Encyclopedia of Genes and Genomes (KEGG) database. We extracted and downloaded metabolic genes from TCGA. Then, we adjusted different mRNA expression levels by ‘sva’ package and selected candidate metabolic genes by R software (version 3.6.1). Differentially expressed genes (DEGs) were screened through ‘limma’ package (version 3.44.1) on R, with the screening criteria false discovery rate (FDR) < 0.05 and |log2-fold change| > 1. Heatmap and volcano were constructed through ‘pheatmap’ package in R.

### Construction of metabolic signature

We used the dataset from TCGA as the training cohort. Univariate cox regression analysis was performed to identify prognostic metabolic genes by ‘survival’ and ‘survminer’ package, with the screening criteria *P* < 0.05. We regarded overall survival (OS) as the primary outcome and genes with HR < 1 were defined as better prognosis. The metabolic risk model was constructed after 1000 resamples by Least Absolute Shrinkage and Selection Operator (LASSO) Cox regression analysis through ‘glmnet’ and ‘survival’ package on R.

### The performance of gene signature

We applied the metabolic risk model to TCGA datasets. Patients were grouped into high- and low-risk groups according to the median risk score. Kaplan–Meier curves were generated by survival analysis through the ‘survival’ package. Risk score curves were drawn using the ‘pheatmap’ package. Next, univariate and multivariate cox proportional hazards analysis were performed. Time-dependent receiver operating characteristic (ROC) curve and the area under the ROC curve (AUC) were used to evaluate the sensitivity and specificity of the model using the ‘survival ROC’ package. For evaluation of the performance of the model, we compared the prediction model with the existing model (TNM stage) by the time-dependent ROC curve^16^. The 1-, 3-, and 5-year ROC curves of the model were plotted and the values of AUC were calculated with packages ‘survival’, ‘survminer’, and ‘survival ROC’ in R. A nomogram was built to visualize the risk model and classic independent risk factors, including age, gender, tumor grade, and TMN stage, to calculate survival rate of cancer patients.

### Validation of metabolic risk model

We investigated extensively in GEO datasets and collected all appropriate datasets to validate the predictive ability of the model. Patients were grouped into high- and low-risk groups and Kaplan–Meier curves were generated by survival analysis through the “survival” package. Risk score curves were drawn using the ‘pheatmap’ package.

### Gene set enrichment analyses

GSEA software (version 4.0.3) for Windows was downloaded from website for functional analyses. KEGG and GO (Gene Ontology) enrichment analyses of the DEGs were performed to identify potential pathways and functions by GSEA.

### WGCNA of DEGs

To assess the inter-correlation of the intensities of the DEGs and the relationship between them and feature data, a weighted gene co-expression network analysis (WGCNA) was performed using the WGCNA R package. Firstly, we conducted quality testing and preprocessing of the data^17,18^. Normalization was performed using ‘preprocessCore’ package, while outliers were removed and missing values were treated by using the ‘impute’ package. After the procedure of preprocessing, we got a reasonable and useful sample set. Secondly, a matrix of adjacencies was built according to the calculated absolute value of the Pearson’s correlation coefficients between each of the gene pairs. Thirdly, the matrix was applied to a network construction and module detection function by building a weighted matrix with a scaling factor-β. Then, the adjacencies were transformed into topological overlap matrix (TOM). Similar modules were merged to trim genes whose correlation with module eigengene was less than the defined threshold (min Module Size of 30 and merge Cut Height of 0.25). Fourthly, we calculated the correlation between clinical information and module eigengenes (MEs) to identify the clinically significant modules. Finally, we calculated the correlation between genes and clinical traits, as well as the correlation between genes and MEs. WGCNA determines highly inter-connected nodes as modules designated with different colors.

### PPI network construction and functional analyses

Protein–protein interaction (PPI) network was constructed by Search Tool for the Retrieval of Interacting Genes (STRING, https://string-db.org/) and the CytoHubba plug-in in the Cytoscape software (version 3.7.1). Then we analyzed the relationships between each core genes and clinical characteristics. The ‘clusterProfiler’, ‘enrichplot’, and ‘ggplot2’ software packages in R were used to perform Gene Ontology (GO) and Kyoto Encyclopedia of Genes and Genomes (KEGG) enrichment analyses. GO has three independent branches: molecular function (MF), biological process (BP), and cellular component (CC). An FDR of < 0.05 was defined as statistically significant.

### Consent for publication

All the authors listed have approved the submission and publication.

## Results

### Data extraction

**Figure 1:**
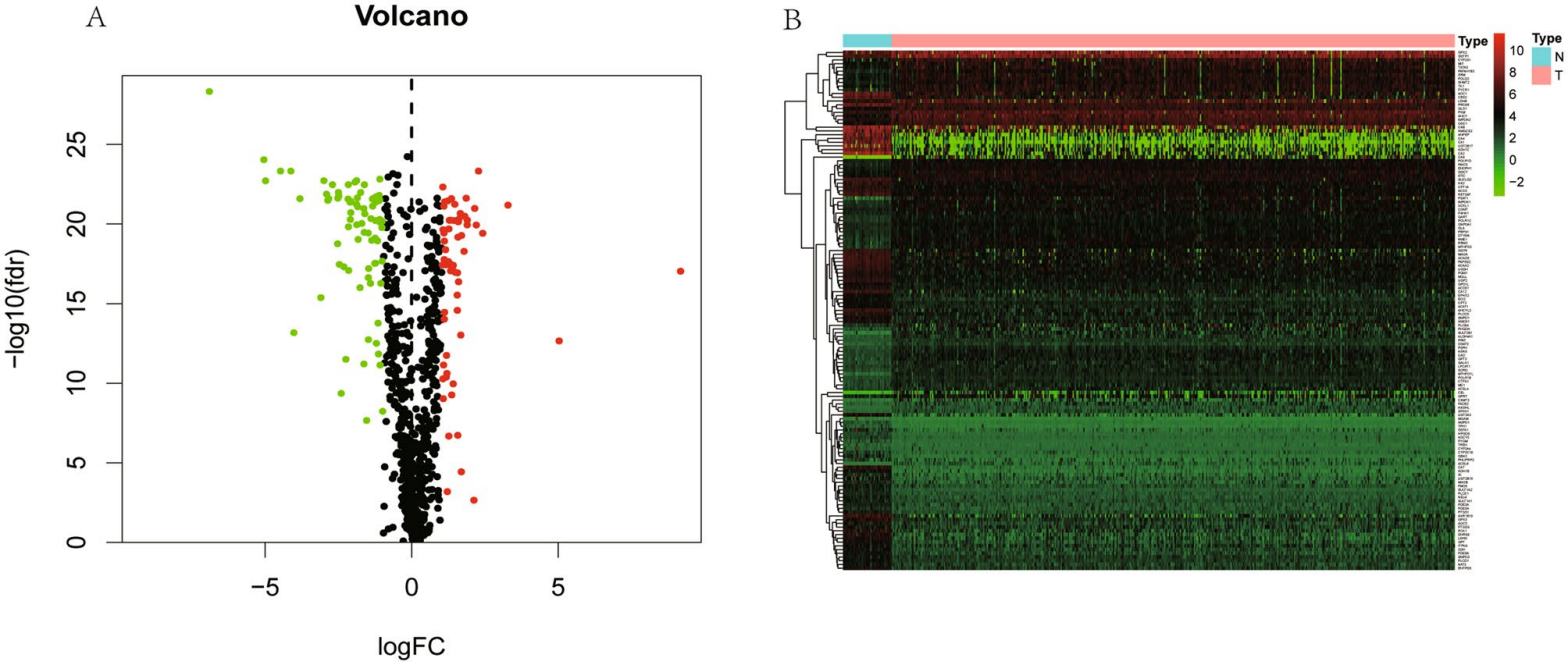
Volcano plot and Heatmap of differentially expressed metabolic genes. (**A**) Volcano plot of differentially expressed metabolic genes. The red points represent high expression genes, the green points represent low expression genes, the black points represent genes with no significant difference (FDR < 0.05, log FC > 1.0). (**B**) Heatmap of differentially expressed metabolic genes. Red indicates that the gene expression is relatively high, green indicates that the gene expression is relatively low, and black indicates no significant changes in gene expression (FDR < 0.05, absolute log FC > 1.0).

We extracted 452 cases and 753 metabolic genes from TCGA database, including 361 up-regulated and 392 down-regulated genes (Supplementary Table 1). Then we downloaded and combined gene expression matrix of the GSE17536, GSE17537, and GSE29621 series and obtained 177 cases. After screening, we identified 139 differentially expressed metabolic genes, including 62 up-regulated and 77 down-regulated genes, which were shown in volcano plot (Fig. 1A) and heatmap (Fig. 1B).

### Construction of metabolic signature

**Figure 2:**
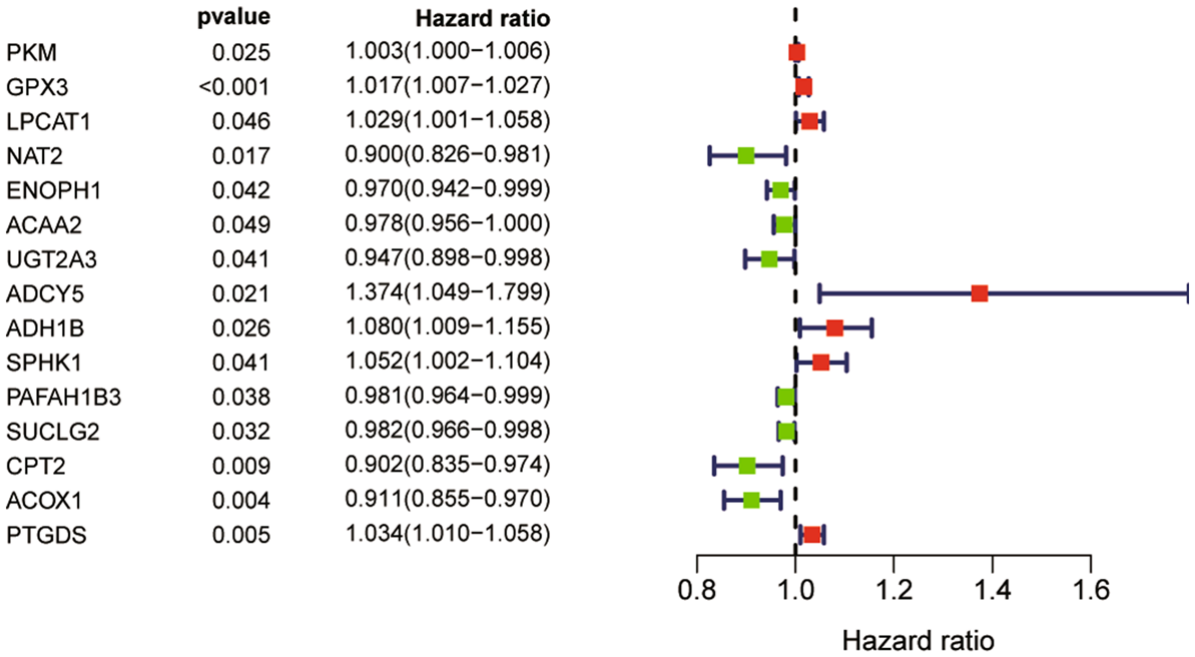
Forest plot of the univariate Cox regression analysis showing the prognostic metabolic genes. Genes with HR < 1 have better overall survival outcomes, while genes with HR > 1 have worse overall survival outcomes.

A total of 15 prognostic genes were dug out by univariate cox regression analysis. Among them, NAT2, ENOPH1, ACAA2, UGT2A3, PAFAH1B3, SUCLG2, CPT2, and ACOX1 were associated with better overall survival outcomes, while PKM, GPX3, LPCAT1, ADCY5, ADH1B, SPHK1, and PTGDS were associated with worse overall survival outcomes (Fig. 2). Then an eleven-gene prognostic model was constructed after 1000 resamples by LASSO penalized Cox regression analysis. Risk score = 0.0011 × expression of PKM + 0.0076 × expression of GPX3 + 0.0052 × expression of LPCAT1 − 0.0060 × expression of ENOPH1 − 0.0044 × expression of ACAA2 − 0.0041 × expression of UGT2A3 + 0.0934 × expression of ADCY5 − 0.0119 × expression of PAFAH1B3 − 0.0578 × expression of CPT2 − 0.0461 × expression of ACOX1 + 0.0102 × expression of PTGDS.

### The performance of gene signature

**Figure 3:**
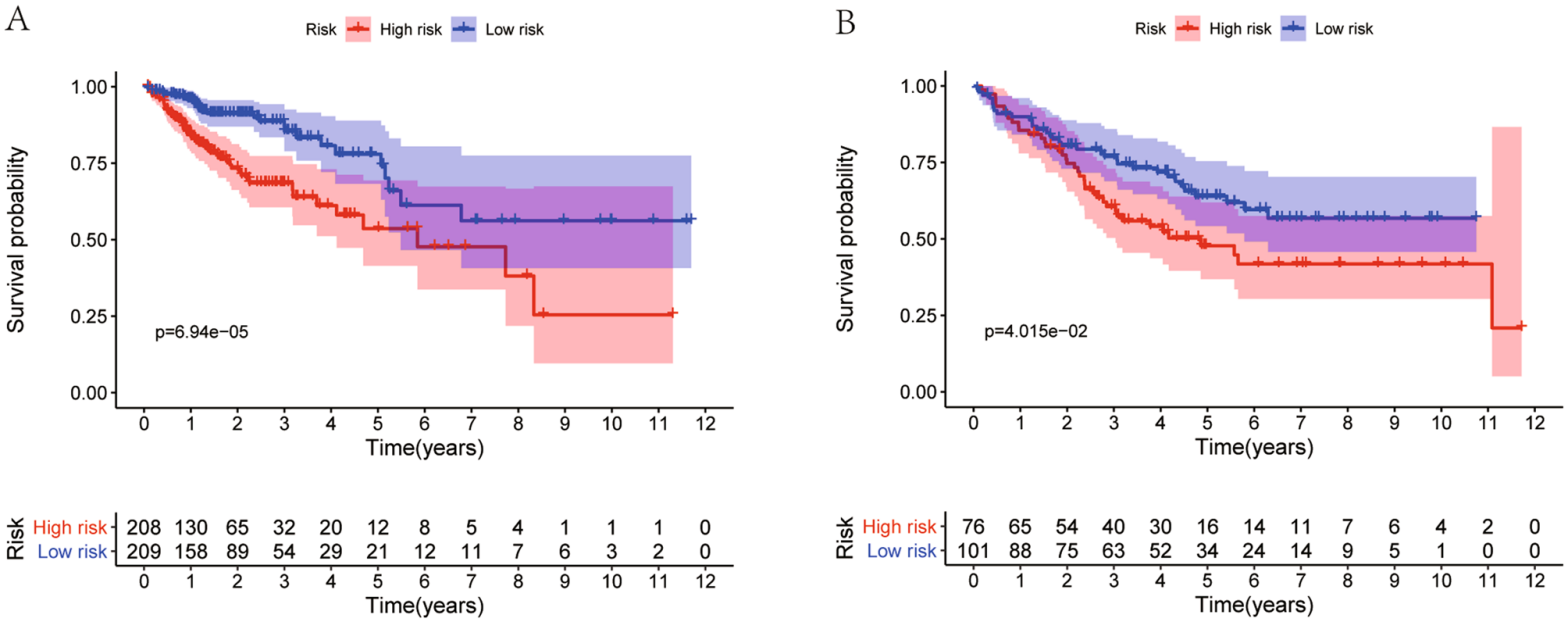
Kaplan–Meier curves of Metabolic Signature. (**A**) Kaplan–Meier curves of gene signature based on TCGA. (**B**) Kaplan–Meier curves of gene signature based on GEO.

**Figure 4:**
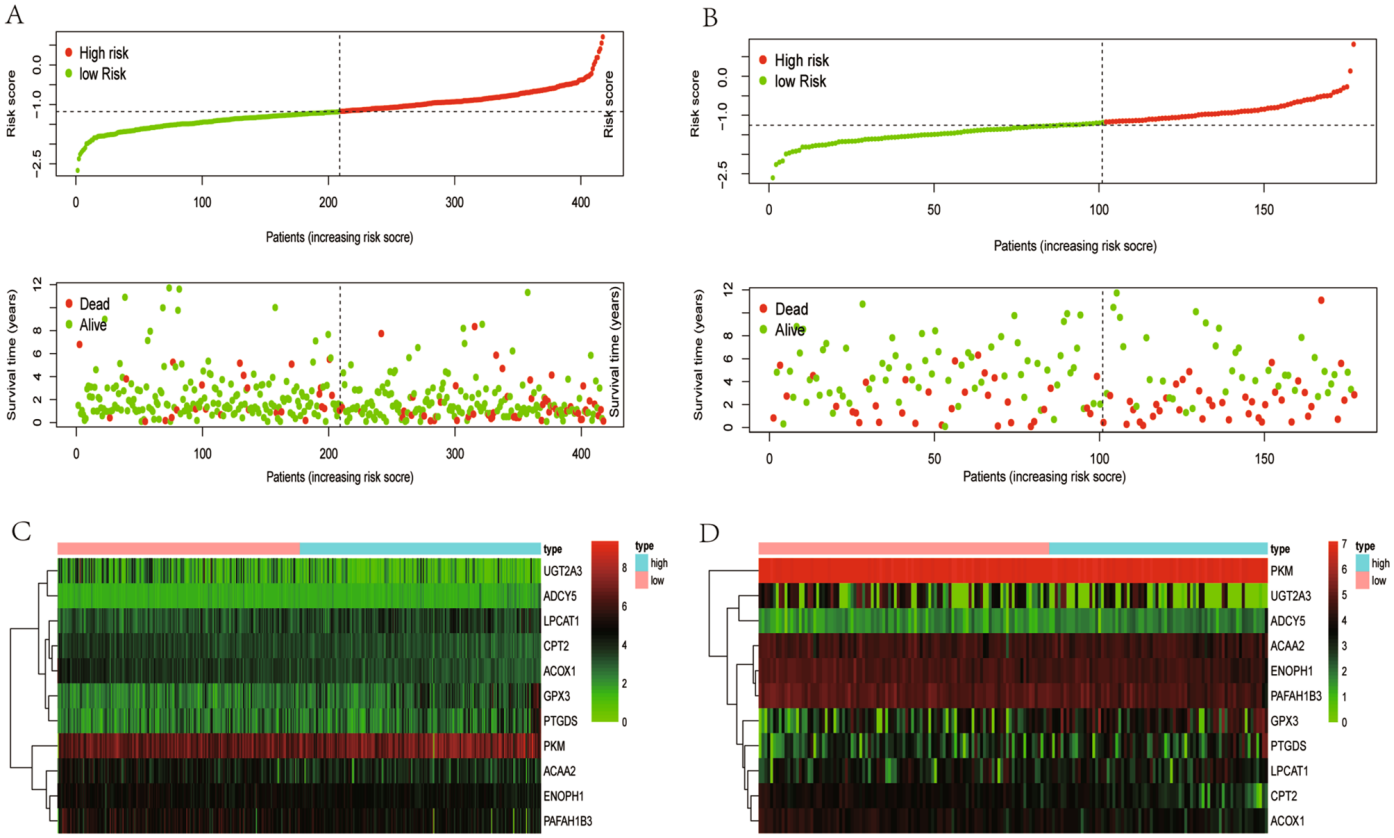
Risk scores of Metabolic Signature. (**A**) Risk score distribution of the eleven -gene signatures based on TCGA database. (**B**) Risk score distribution of the eleven-gene signatures based on GEO database. (**C**) Heatmaps of the differential expression of metabolic genes in high-risk and low-risk groups based on TCGA database. (**D**) Heatmaps of the differential expression of metabolic genes in high-risk and low-risk groups based on GEO database.

**Figure 5:**
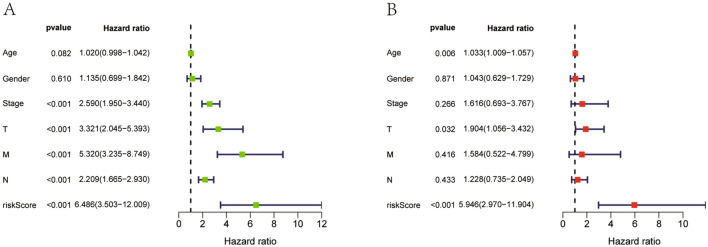
Univariate and Multivariate Cox analysis. (**A**) Prognostic value detection of the gene signature via univariate survival-related analysis in TCGA. (**B**) Prognostic value detection of the gene signature via multivariate survival-related analysis in TCGA.

**Figure 6:**
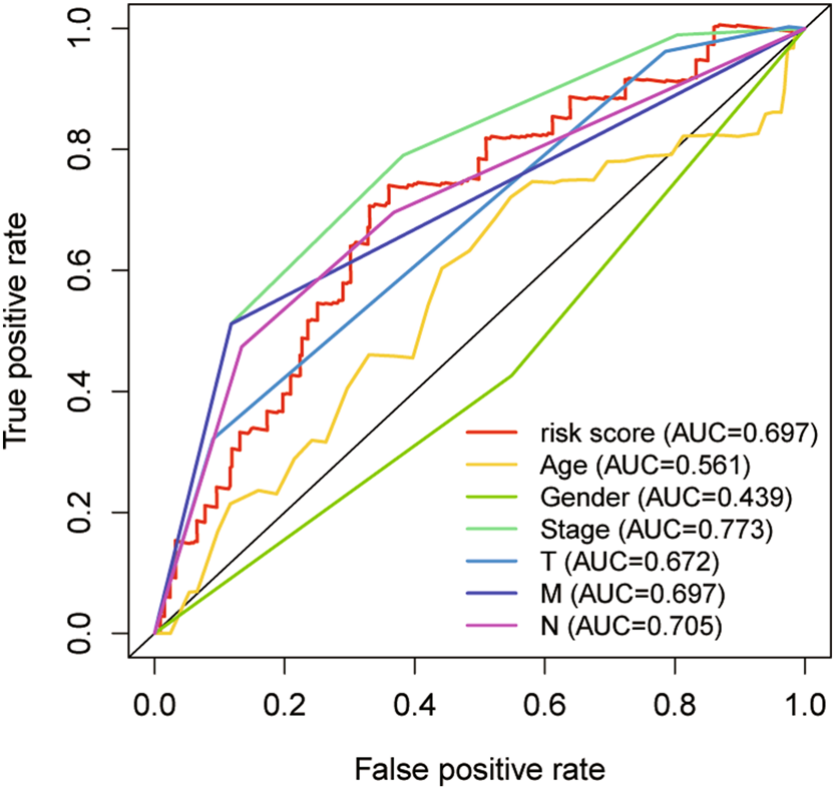
Time-dependent ROC analysis of Metabolic Signature in TCGA cohorts. ROC, receiver operating characteristic; AUC, area under the ROC curve.

**Figure 7:**
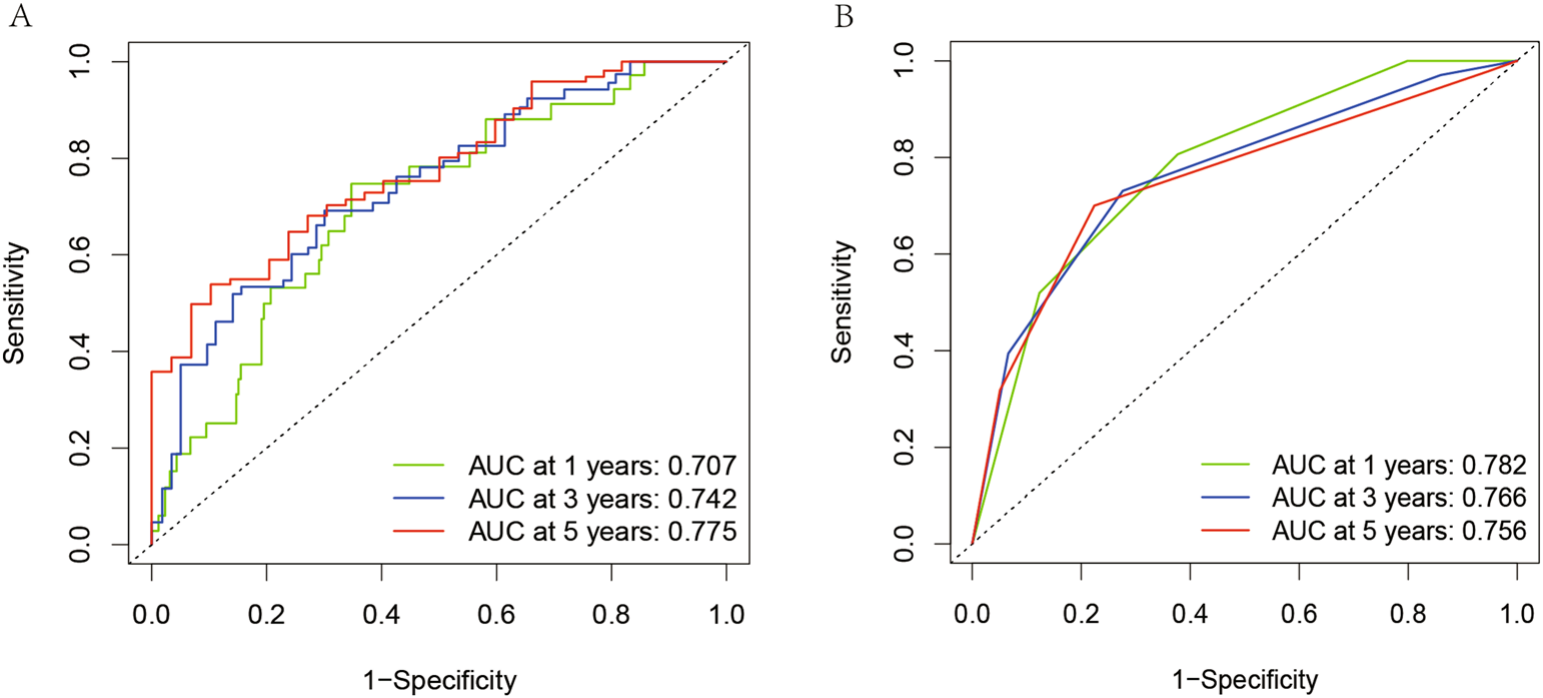
The comparison of this model with TNM stage by the time-dependent ROC curve and AUC of 1, 3, and 5-year. (**A**) For our predict model, the AUC was 0.707 (1-year), 0.742 (3-year) and 0.775 (5-year), respectively. (**B**) For TMN stage, the AUC was 0.782 (1-year), 0.766 (3-year) and 0.758 (5-year), respectively.

**Figure 8:**
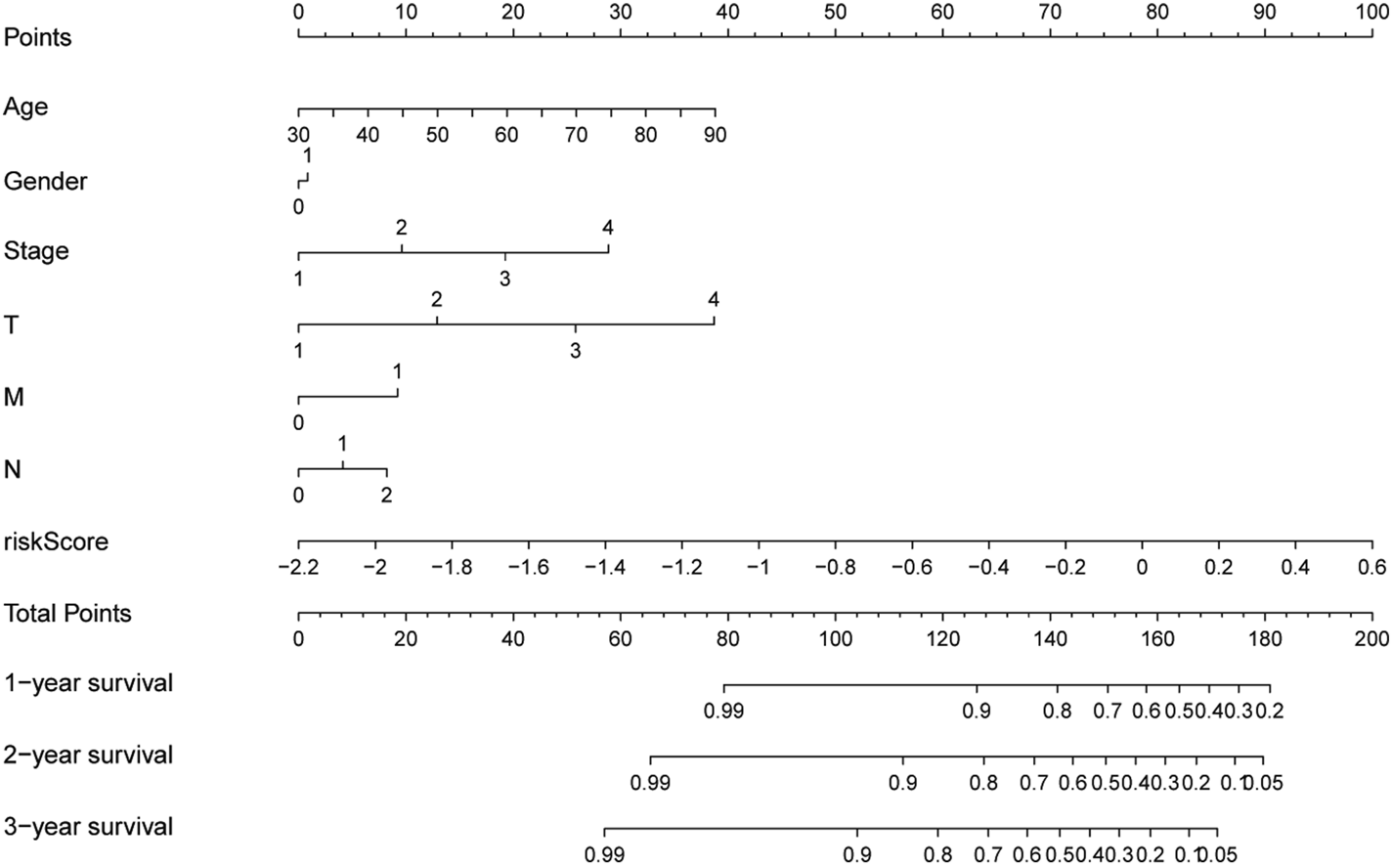
Nomogram was built to predict overall survival for colon cancer patients.

Patients were grouped into high- and low-risk groups according to the median risk score. In the high, patients had a lower OS according to Kaplan–Meier curves (*P* < 0.05, Fig. 3A) and risk score distribution than in the low (Fig. 4A). The differential expression of metabolic genes in high- and low-risk groups were shown in heatmaps (Fig. 4C). Univariate Cox analysis showed that the risk of poor prognosis elevated with the risk score increasing (Fig. 5A). Multivariate Cox regression analysis demonstrated that the risk score could be an independent risk factor for OS (Fig. 5B). The AUC was 0.697 according to the ROC, which was higher than age (AUC = 0.561), gender (AUC = 0.439), and T stage (AUC = 0.672) (Fig. 6). We compared the model with TNM stage by the time-dependent ROC curve and the values of AUC. For our predict model, the AUC was 0.707 (1-year), 0.742 (3-year) and 0.775 (5-year), respectively (Fig. 7A). For TMN stage, the AUC was 0.782 (1-year), 0.766 (3-year) and 0.758 (5-year), respectively (Fig. 7B). Nomogram was formed of age, gender, tumor stage, TNM stage and the risk score as well (Fig. 8).

### Validation of metabolic signature

We adjusted and combined GSE17536, GSE17537, and GSE29621 as validation dataset to reanalyze. Patients were grouped into high- and low-risk groups according to the median risk score. In the high, patients had a lower OS according to Kaplan–Meier curves (*P* < 0.05, Fig. 3B) and risk score distribution than in the low (Fig. 4B). The differential expression of metabolic genes in high- and low-risk groups were shown in heatmaps (Fig. 4D).

### GSEA

**Figure 9:**
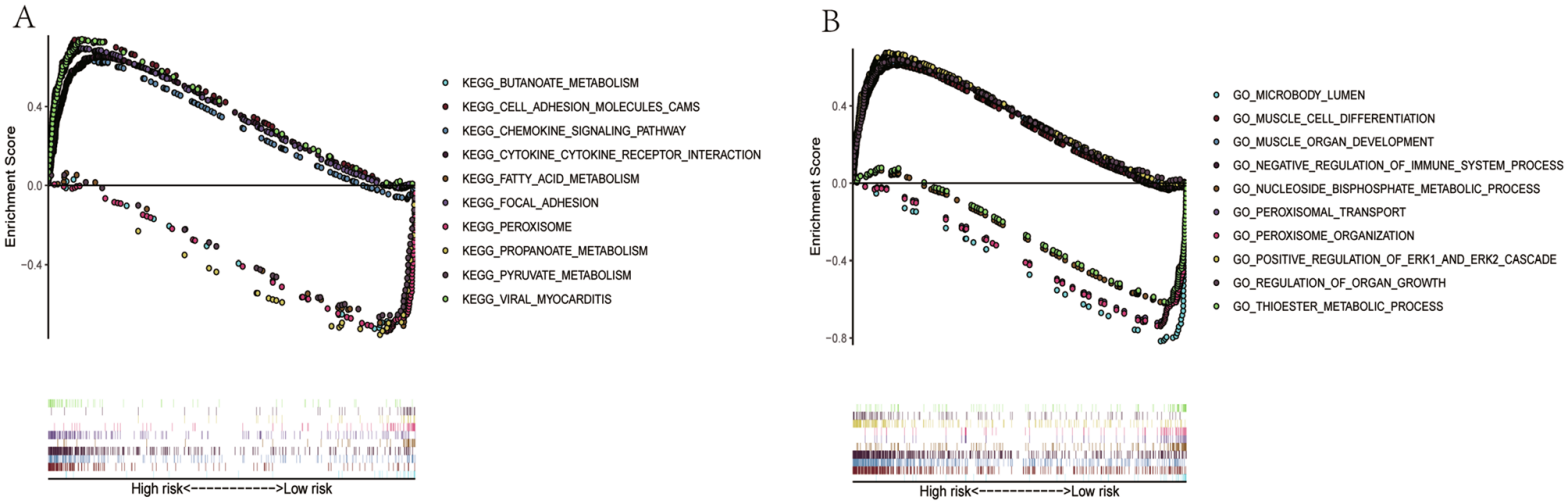
KEGG pathways and GO enrichment analyses by GSEA. (**A**) Top 10 representative KEGG pathways. (**B**) Top 10 representative GO terms.

The results of GSEA showed that significantly metabolism-related pathways were enriched in the low-risk group. KEGG analysis demonstrated that most of the enriched pathways focused on fatty acids (FAs) metabolism, pyruvate metabolism, propanoate metabolism, and butanoate metabolism pathways (Fig. 9A). GO analysis showed that genes were mainly enriched in nucleoside bisphosphate metabolic process, and thioester metabolic process (Fig. 9B). In addition, there were several famous cancer-related pathways, such as cell adhesion molecules (CAMs), cytokine-cytokine receptor interaction, peroxisome, peroxisomal transport, peroxisome organization.

### WGCNA of DEGs

**Figure 10:**
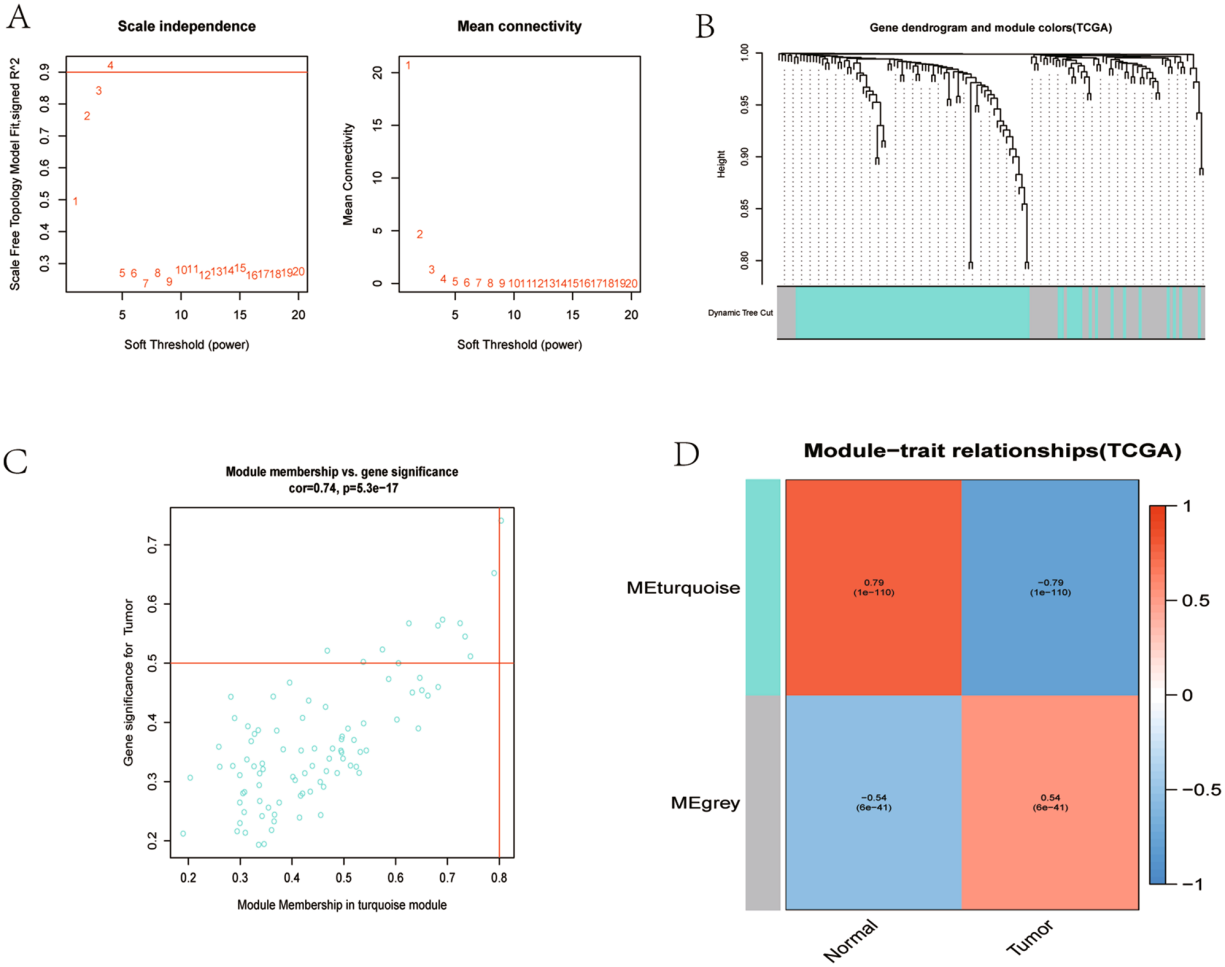
A weighted gene co-expression network analysis (WGCNA) of the DEGs. (**A**) A scaling factor beta determination based on the scale-free topology criterion. (**B**) Hierarchical clustering of genes in significant modules. The colors are assigned to each module by the Dynamic Tree Cut algorithm. (**C**) Scatterplot shows a highly significant correlation between gene significant (GS) versus module membership (MM) in the turquoise module with tumor. (**D**) Heatmap shows most significant and correlated module with tumor was blue module.

Figure 10A shows the determination of β parameter based on the description in the WGCNA manual. We established two modules based on genes expression pattern via average linkage clustering (Fig. 10B). Modules in gene dendrogram are shown in different colors and based on dynamic branch cutting algorithm underneath row color assigns the modules membership. Figure 10C shows a highly significant correlation between gene significant (GS) versus module membership (MM) in the turquoise module with tumor, and Fig. 10D shows the most significant and correlated module with tumor was blue module.

### PPI network construction and functional analyses

**Figure 11:**
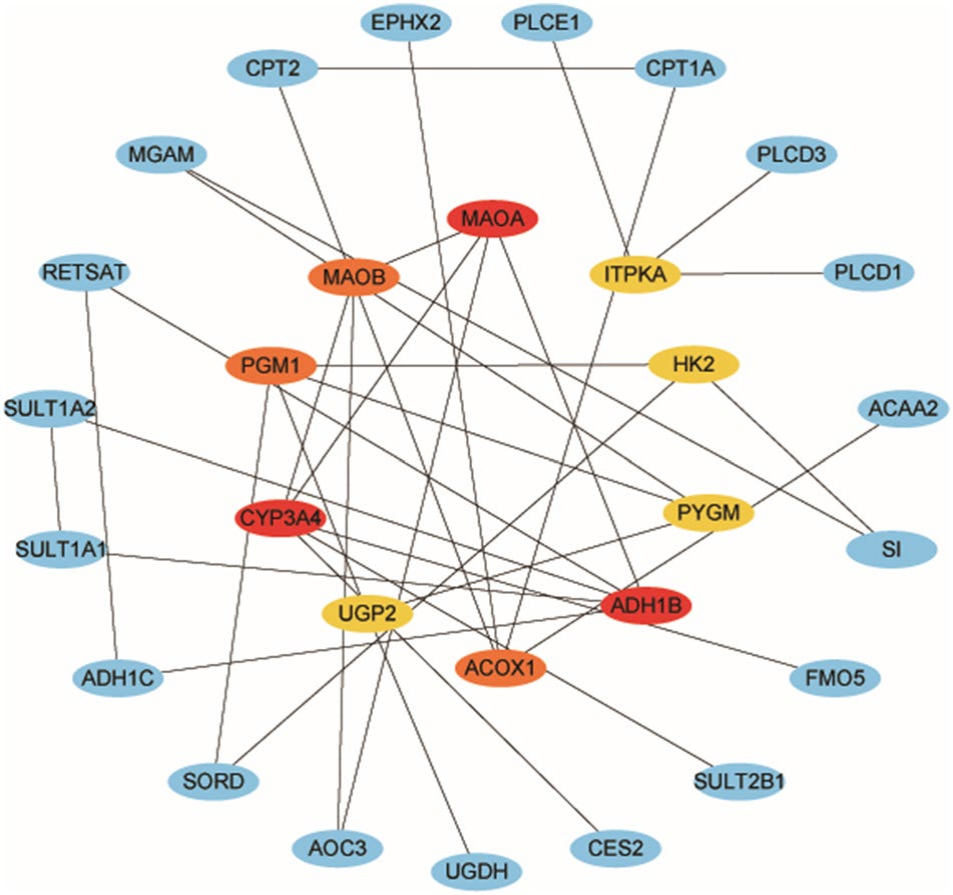
PPI network of the top 10 interconnected genes in the blue modules.

We selected the genes with high GS and MM from blue module based on the co-expression network to constructed PPI networks and highlighted the high connectivity genes. The PPI network showed the top 10 central metabolic genes in colon cancer (Fig. 11). Of all the central genes, PYGM was correlated with gender, T stage, and N stage, while ACOX1, ADH1B, and MAOB were significantly correlated with N stage (Supplementary Fig. 1). GO enrichment results show the top 10 BP terms, 10 CC terms and 10 MF terms, The KEGG enrichment results show the 30 paths (Supplementary Fig. 2).

## Discussion

In this study, we established a metabolic risk model to predict the prognosis of colon cancer based on TCGA and verified by GEO. Firstly, we identified differentially expressed metabolic genes from TCGA and constructed a prognostic model by LASSO. The gene signature has been proved to have an excellent ability to predict prognosis by Kaplan–Meier analysis, time-dependent receiver operating characteristic (ROC), risk score, univariate and multivariate cox regression analysis based on TCGA. Then, we verified the model by Kaplan–Meier curves and risk score based on GEO. Finally, we performed WGCNA, PPI network and functional analyses of DEGs.

Colon cancer is a malignant tumor with poor prognosis, so novel prognostic biomarkers are urgently needed. In recent years, there have been many publications and studies on the risk prediction model for the prognosis of colon cancer, including circulating tumor DNA^19^, mRNA^20^, miRNA^21^, and clinical characters^22^. Energy metabolism is the basis of proliferation and invasion for tumor cell, and most tumor cells show deviation from the normal energy metabolism state. The prognostic models based on metabolic genes have been established in rectal cancer^23^, lung adenocarcinoma^24^, hepatocellular carcinoma^25^, and acute myelogenous leukemia^26^. But it has not been used in colon cancer. In this study, we identified eleven prognostic genes involved in metabolism, which revealed the metabolism alterations in colon cancer. Theses metabolic genes can be used as potential biomarkers and therapeutic target for colon cancer patients.

In this model, the expression of ENOPH1, ACAA2, UGT2A3, PAFAH1B3, CPT2, and ACOX1 predicted better prognosis, whereas the expression of PKM, GPX3, LPCAT1, ADCY5 and PTGDS predicted worse prognosis. Most of the genes have been proved to take part in pathogenesis, progression and prognosis of cancers. We compared this metabolic risk model with other metabolic signatures in previous studies and found that PAFAH1B3 is a good prognostic gene in myelogenous leukemia^26^ while LPCAT1 is a risky prognostic gene in hepatocellular carcinoma^27^, which has the same function in colon cancer. Platelet activating factor acetyl hydrolase 1B3 (PAFAH1B3) is involved in ether lipid metabolism, and was reported to play an important role in tumorigenesis and aggressiveness in many different cancers^28^. Blockers of PAFAH1B3 could heightened levels of tumor-suppressing lipids, then impairs pathogenicity of different cancers, including breast, ovarian, melanoma, and prostate cancer^29^. LPCAT1, an important subtype belongs to the LPCAT subtypes, is a cytosolic enzyme which converts lysophosphatidylcholine (LPC) to phosphatidylcholine (PC). To date, the over-expression of LPCAT1 has been reported in the contribution of the progression, metastasis, and recurrence of multiple types of cancers, including cell renal cell carcinoma^30^, gastric cancer^31^, breast cancer^32^, oral squamous cell carcinoma^33^, and hepatocellular carcinoma^34^.

Enolase-phosphatase 1 (ENOPH1), a bifunctional enolase-dephosphorylase enzyme^35^, is required for polyamine biosynthesis^36^. Previous studies showed that overexpression of ENOPH1 remarkably promoted cell migration and invasiveness, whereas the downregulation of ENOPH1 significantly impaired cell migration and invasiveness^37^. Acetyl-Coenzyme A acyltransferase 2 (ACAA2) exerts an effect on β-oxidation of fatty acid, which then provide energy^38^. Previous showed that ACAA2 could abolished the apoptosis in human hepatocellular carcinoma^39^, and played a vital role in the metabolism processes in gliomas. The higher expression of ACAA2 was associated with better prognosis of colorectal cancer^40^. Carnitine palmitoyl transferase 2 (CPT2), a rate-limiting enzyme for mitochondrial fatty acid transportation, had been proved to be a protective prognostic gene for colorectal cancer^41^, which is in accordance with our results. Peroxisomal Acyl-Coa Oxidase 1 (ACOX1) is the rate-limiting enzyme in fatty acid β-oxidation. The inhibitor of ACOX1 is SIRT1, which has been proved to prevent oxidative damage and is downregulated in liver cancerr^42,43^, suppresses colorectal cancer metastasis by transcriptional repression of miR-15b-5p^44^.

Pyruvate kinase M (PKM), a metabolic regulator, participated in both glycolytic and non-glycolytic pathways. PKM also acts as protein kinase, which shifts the glucose metabolism from the respiratory chain to aerobic glycolysis in tumor cells. PKM2 is upregulated in most cancer types, and contributes to tumorigenesis^45^, which suggested that it could act as a remarkable therapeutic target^46^. The glutathione peroxidases-3 (GPX3), a selenocysteine-containing redox enzyme, took part in reactive oxygen species signaling and immunomodulatory^47^. Previous studies suggested that GPX3 prevented the colitis-associated carcinoma by immunomodulation^48^. But the correlation between GPX and prognosis of colon cancer is not clear. The tumor suppressive effect of prostaglandin D2 (PGD2) on testicular cancer and gastric cancer has been confirmed^49^. However, the correlation between PGD2 and colon cancer is unclear.

The gene signature has been proved to have an excellent ability to predict prognosis by Kaplan–Meier analysis, ROC and AUC, univariate and multivariate cox regression analysis based on TCGA. In addition, we compared the model with TNM stage by the ROC curve and the values of AUC. The results showed that our predict model was more accurate in the prediction of the 5-year survival rate (AUC = 0.775 vs 0.758). Besides, genetic testing is more accurate and convenient, without the need for surgery, so the prediction model is much more advanced for clinicians and researchers.

It is acknowledged that diverse factors affect tumor development and prognosis, including clinic-pathological diagnosis, immunology regulations, gut microbiome, and metabolism of glucose and lipids^50^. The TNM classification system is the major pathological staging method, which is the most widely used staging system for clinical decision making^51,52^. Increasing evidence indicates the key role of systemic immunity in tumor progression and outcome^53–55^. Recent studies highlight the role of the microbiota composition in intestinal inflammation in mice and humans, which has been linked to colorectal cancer^56^. There have been many publications and studies on the risk prediction model for the prognosis of colon cancer based on immune genes^57–62^. The abnormal metabolism of tumor glucose and lipids is an important part of tumor metabolic reprogramming, which is closely related to tumor occurrence, development, and prognois^50^. In this study, the functional analyses revealed many metabolic pathways related to prognosis of colon cancer. In one hand, the results validated the close association between metabolic systems and colon cancer. GSEA analyses showed most significantly enriched pathways focused on fatty acid metabolism, butanoate metabolism, propanoate metabolism and pyruvate metabolism pathways. Most of the metabolic genes in the model are fatty metabolism related genes, including PAFAH1B3, LPCAT1, ACAA2, ACOX1, and CPT2.The top four significant genes in PPI network ACOX1, CPT2, ACAA2, and PKM were involved in lipid metabolism or glycolysis. Changes of glucose and lipid metabolism is one of the key features of cancer cells because cell proliferation requires increased lipid biosynthesis, and bioactive molecules produced by lipid catabolism will act as signal molecules to regulate cancer metastasis^63,64^. Increased glycolysis is the main source of energy supply in cancer cells that use this metabolic pathway for ATP generation. Fatty acids are the preferred substrates for energy storage, mainly in the form of triglyceride. Availability of fatty acids in the blood and peripheral tissues depends on lifestyle and diet and is likely to be altered in patients who are obese or have metabolic syndrome. It is important to keep a healthy diet, including less high saturated fatty acids and lose weight.

In the other hand, most of the metabolic pathways were enriched in the low-risk patients, while the non-metabolic pathways were enriched in the high-risk patients. As a result, metabolic therapy may be more suitable for low‐risk patients, while high‐risk patients might benefit more from nonmetabolic therapy. Previous clinical studies have shown the effectiveness of glycolytic therapy to suppress cancer progression^65–67^. So far, the application of metabolism in malignant tumors includes diagnosis, identification of biomarkers and tumor metabolic processes. In the future, colon cancer patients might benefit from metabolic-related therapy and management.

However, there were some limitations in this study. Firstly, there was no functional experiment in the real world. Secondly, deep learning is widely used for intelligence medicine to assistant disease diagnosis and risk prediction, like imaging data, genomic or transcriptomic data^68^, but we adopted Lasso and multivariate Cox regression to build a risk model which may result in some limitations. Thirdly, we didn’t test disease-associated modules for preservation by use of an independent dataset, which may lead to reliability decreases^17,18^. Moreover, it is relatively weak to take only metabolic genes into prognostic model because much more complicated mechanisms contributed to colon cancer.

## Conclusions

We constructed and validated a metabolic risk model to predict the prognosis of colon cancer based on TCGA and GEO database. Metabolic-related therapied and managements could be considered for colon cancer patients. However, validation and functional experiments of the metabolic risk model are indispensable.

## Supplementary Information

Supplementary Legends.

Supplementary Figure 1.

Supplementary Figure 2.

Supplementary Tables.

## Data Availability

The datasets and analyses during the current study are available in TCGA and GEO database.

## References

[1] Benson AB, Venook AP, Al-Hawary MM (2018). NCCN guidelines insights: Colon cancer, version 2.2018. J. Natl. Compr. Cancer Netw..

[2] Siegel RL, Miller KD, Goding SA (2020). Colorectal cancer statistics, 2020. CA Cancer J. Clin..

[3] Siegel RL, Fedewa SA, Anderson WF (2017). Colorectal cancer incidence patterns in the United States, 1974–2013. J. Natl. Cancer Inst..

[4] Islami F, Goding SA, Miller KD (2018). Proportion and number of cancer cases and deaths attributable to potentially modifiable risk factors in the United States. CA Cancer J. Clin..

[5] Winawer SJ, Zauber AG (2002). The advanced adenoma as the primary target of screening. Gastrointest. Endosc. Clin. N. Am..

[6] Edwards BK, Ward E, Kohler BA (2010). Annual report to the nation on the status of cancer, 1975–2006, featuring colorectal cancer trends and impact of interventions (risk factors, screening, and treatment) to reduce future rates. Cancer-Am. Cancer Soc..

[7] Porta C, Sica A, Riboldi E (2018). Tumor-associated myeloid cells: New understandings on their metabolic regulation and their influence in cancer immunotherapy. FEBS J..

[8] Qu W, Oya S, Lieberman BP (2012). Preparation and characterization of L-[5-11C]-glutamine for metabolic imaging of tumors. J. Nucl. Med..

[9] Hanahan D, Weinberg RA (2011). Hallmarks of cancer: The next generation. Cell.

[10] Demirkol Canlı S, Seza EG, Sheraj I (2020). Evaluation of an aldo-keto reductase gene signature with prognostic significance in colon cancer via activation of epithelial to mesenchymal transition and the p70S6K pathway. Carcinogenesis.

[11] DeBerardinis RJ, Lum JJ, Hatzivassiliou G, Thompson CB (2008). The biology of cancer: Metabolic reprogramming fuels cell growth and proliferation. Cell Metab..

[12] Tabe Y, Lorenzi PL, Konopleva M (2019). Amino acid metabolism in hematologic malignancies and the era of targeted therapy. Blood.

[13] Chen W, Gao C, Liu Y (2020). Bioinformatics analysis of prognostic miRNA signature and potential critical genes in colon cancer. Front. Genet..

[14] Zhang Z, Zhang S, Yang J (2020). Integrated transcriptomic and metabolomic analyses to characterize the anti-cancer effects of (-)-epigallocatechin-3-gallate in human colon cancer cells. Toxicol. Appl. Pharm..

[15] Gharib E, Nasri Nasrabadi P, Reza ZM (2020). miR-497-5p mediates starvation-induced death in colon cancer cells by targeting acyl-CoA synthetase-5 and modulation of lipid metabolism. J. Cell. Physiol..

[16] Yu H, Pan R, Qi Y (2020). LEPR hypomethylation is significantly associated with gastric cancer in males. Exp. Mol. Pathol..

[17] Chen J, Zhao X, Cui L (2020). Genetic regulatory subnetworks and key regulating genes in rat hippocampus perturbed by prenatal malnutrition: Implications for major brain disorders. Aging.

[18] Li H, Wang X, Lu X (2019). Co-expression network analysis identified hub genes critical to triglyceride and free fatty acid metabolism as key regulators of age-related vascular dysfunction in mice. Aging.

[19] Luo H, Zhao Q, Wei W (2020). Circulating tumor DNA methylation profiles enable early diagnosis, prognosis prediction, and screening for colorectal cancer. Sci. Transl. Med..

[20] Zuo S, Dai G, Ren X (2019). Identification of a 6-gene signature predicting prognosis for colorectal cancer. Cancer Cell Int..

[21] Di Z, Di M, Fu W (2020). Integrated analysis identifies a nine-microRNA signature biomarker for diagnosis and prognosis in colorectal cancer. Front. Genet..

[22] Boakye D, Jansen L, Schneider M (2019). Personalizing the prediction of colorectal cancer prognosis by incorporating comorbidities and functional status into prognostic nomograms. Cancers.

[23] Zhang ZY, Yao QZ, Liu HY (2020). Metabolic reprogramming-associated genes predict overall survival for rectal cancer. J. Cell. Mol. Med..

[24] Zhang S, Lu Y, Liu Z (2020). Identification six metabolic genes as potential biomarkers for lung adenocarcinoma. J. Comput. Biol..

[25] Liu GM, Xie WX, Zhang CY, Xu JW (2020). Identification of a four-gene metabolic signature predicting overall survival for hepatocellular carcinoma. J. Cell. Physiol..

[26] Wang Y, Hu F, Li JY (2020). Systematic construction and validation of a metabolic risk model for prognostic prediction in acute myelogenous leukemia. Front. Oncol..

[27] Zhu Z, Li L, Xu J (2020). Comprehensive analysis reveals a metabolic ten-gene signature in hepatocellular carcinoma. PeerJ..

[28] Xu J, Zang Y, Cao S, Lei D, Pan X (2019). Aberrant expression of PAFAH1B3 associates with poor prognosis and affects proliferation and aggressiveness in hypopharyngeal squamous cell carcinoma. Oncotargets Ther..

[29] Kohnz RA, Mulvihill MM, Chang JW (2015). Activity-based protein profiling of oncogene-driven changes in metabolism reveals broad dysregulation of PAFAH1B2 and 1B3 in cancer. ACS Chem. Biol..

[30] Du Y, Wang Q, Zhang X (2017). Lysophosphatidylcholine acyltransferase 1 upregulation and concomitant phospholipid alterations in clear cell renal cell carcinoma. J. Exp. Clin. Cancer Res..

[31] Uehara T, Kikuchi H, Miyazaki S (2016). Overexpression of lysophosphatidylcholine acyltransferase 1 and concomitant lipid alterations in gastric cancer. Ann. Surg. Oncol..

[32] Abdelzaher E, Mostafa MF (2015). Lysophosphatidylcholine acyltransferase 1 (LPCAT1) upregulation in breast carcinoma contributes to tumor progression and predicts early tumor recurrence. Tumour Biol..

[33] Shida-Sakazume T, Endo-Sakamoto Y, Unozawa M (2015). Lysophosphatidylcholine acyltransferase1 overexpression promotes oral squamous cell carcinoma progression via enhanced biosynthesis of platelet-activating factor. PLoS ONE.

[34] Morita Y, Sakaguchi T, Ikegami K (2013). Lysophosphatidylcholine acyltransferase 1 altered phospholipid composition and regulated hepatoma progression. J. Hepatol..

[35] Wang H, Pang H, Bartlam M, Rao Z (2005). Crystal structure of human E1 enzyme and its complex with a substrate analog reveals the mechanism of its phosphatase/enolase activity. J. Mol. Biol..

[36] Sauter M, Moffatt B, Saechao MC, Hell R, Wirtz M (2013). Methionine salvage and S-adenosylmethionine: Essential links between sulfur, ethylene and polyamine biosynthesis. Biochem. J..

[37] Zhuang H, Qiang Z, Shao X (2019). Integration of metabolomics and expression of enolase-phosphatase 1 links to hepatocellular carcinoma progression. Theranostics..

[38] Kuhajda FP (2000). Fatty-acid synthase and human cancer: New perspectives on its role in tumor biology. Nutrition.

[39] Cao W, Liu N, Tang S (2008). Acetyl-Coenzyme A acyltransferase 2 attenuates the apoptotic effects of BNIP3 in two human cell lines. Biochim. Biophys. Acta.

[40] Guo H, Zeng W, Feng L (2017). Integrated transcriptomic analysis of distance-related field cancerization in rectal cancer patients. Oncotarget.

[41] Liu X, Bing Z, Wu J (2020). Integrative gene expression profiling analysis to investigate potential prognostic biomarkers for colorectal cancer. Med. Sci. Monit..

[42] Huang J, Viswakarma N, Yu S (2011). Progressive endoplasmic reticulum stress contributes to hepatocarcinogenesis in fatty acyl-CoA oxidase 1-deficient mice. Am. J. Pathol..

[43] Chen X, Tian M, Sun R (2018). SIRT5 inhibits peroxisomal ACOX1 to prevent oxidative damage and is downregulated in liver cancer. Embo Rep..

[44] Sun L, Zhi Z, Chen L (2017). SIRT1 suppresses colorectal cancer metastasis by transcriptional repression of miR-15b-5p. Cancer Lett..

[45] Graziano F, Ruzzo A, Giacomini E (2017). Glycolysis gene expression analysis and selective metabolic advantage in the clinical progression of colorectal cancer. Pharmacogenomics J..

[46] Zahra K, Dey T, Ashish, Mishra SP, Pandey U (2020). Pyruvate kinase M2 and cancer: The role of PKM2 in promoting tumorigenesis. Front. Oncol..

[47] Olson GE, Whitin JC, Hill KE (2010). Extracellular glutathione peroxidase (Gpx3) binds specifically to basement membranes of mouse renal cortex tubule cells. Am. J. Physiol. Renal Physiol..

[48] Barrett CW, Ning W, Chen X (2013). Tumor suppressor function of the plasma glutathione peroxidase gpx3 in colitis-associated carcinoma. Cancer Res..

[49] Zhang B, Bie Q, Wu P (2018). PGD2/PTGDR2 signaling restricts the self-renewal and tumorigenesis of gastric cancer. Stem Cells (Dayton, Ohio).

[50] Heske CM (2019). Beyond energy metabolism: Exploiting the additional roles of NAMPT for cancer therapy. Front. Oncol..

[51] Balch CM, Soong SJ, Gershenwald JE (2001). Prognostic factors analysis of 17,600 melanoma patients: Validation of the American Joint Committee on Cancer melanoma staging system. J. Clin. Oncol..

[52] Edge SB, Compton CC (2010). The American Joint Committee on Cancer: The 7th edition of the AJCC cancer staging manual and the future of TNM. Ann. Surg. Oncol..

[53] Parvy JP, Yu Y, Dostalova A (2019). The antimicrobial peptide defensin cooperates with tumour necrosis factor to drive tumour cell death in Drosophila. Elife..

[54] Angelova M, Charoentong P, Hackl H (2015). Characterization of the immunophenotypes and antigenomes of colorectal cancers reveals distinct tumor escape mechanisms and novel targets for immunotherapy. Genome Biol..

[55] Lee N, Zakka LR, Mihm MJ, Schatton T (2016). Tumour-infiltrating lymphocytes in melanoma prognosis and cancer immunotherapy. Pathology.

[56] Zheng S, Zhao T, Yuan S (2019). Immunodeficiency promotes adaptive alterations of host gut microbiome: An observational metagenomic study in mice. Front. Microbiol..

[57] Zhou R, Zhang J, Zeng D (2019). Immune cell infiltration as a biomarker for the diagnosis and prognosis of stage I–III colon cancer. Cancer Immunol. Immunother..

[58] Tian X, Zhu X, Meng W (2020). A 12-immune cell signature to predict relapse and guide chemotherapy for stage II colorectal cancer. Aging.

[59] Dai S, Xu S, Ye Y, Ding K (2020). Identification of an immune-related gene signature to improve prognosis prediction in colorectal cancer patients. Front. Genet..

[60] Chen H, Luo J, Guo J (2020). Development and validation of a five-immune gene prognostic risk model in colon cancer. BMC Cancer.

[61] Lu Y, Zhou X, Liu Z (2020). Assessment for risk status of colorectal cancer patients: A novel prediction model based on immune-related genes. DNA Cell Biol..

[62] Wen S, He L, Zhong Z, Mi H, Liu F (2020). Prognostic model of colorectal cancer constructed by eight immune-related genes. Front. Mol. Biosci..

[63] Huang C, Freter C (2015). Lipid metabolism, apoptosis and cancer therapy. Int. J. Mol. Sci..

[64] Santos CR, Schulze A (2012). Lipid metabolism in cancer. FEBS J..

[65] Ganapathy-Kanniappan S, Geschwind JH (2013). Tumor glycolysis as a target for cancer therapy: Progress and prospects. Mol. Cancer..

[66] Gill KS, Fernandes P, O'Donovan TR (2016). Glycolysis inhibition as a cancer treatment and its role in an anti-tumour immune response. Biochem. Biophys. Acta..

[67] Abdel-Wahab AF, Mahmoud W, Al-Harizy RM (2019). Targeting glucose metabolism to suppress cancer progression: Prospective of anti-glycolytic cancer therapy. Pharmacol. Res..

[68] Liu M, Li F, Yan H (2020). A multi-model deep convolutional neural network for automatic hippocampus segmentation and classification in Alzheimer's disease. Neuroimage.

