# Supplementary material for: Construction and validation of a metabolic risk model predicting prognosis of colon cancer
Source: Sci Rep. 2021 Mar 25;11:6837. doi: 10.1038/s41598-021-86286-z (PMC7994414; doi:10.1038/s41598-021-86286-z)
Supplement: Supplementary file 1 — Supplementary Legends. [file 41598_2021_86286_MOESM1_ESM.docx]

Supplementary Figure 1. The relationships between core genes and clinical characteristics.

Supplementary Figure 2. GO and KEGG enrichment results. (A.) GO enrichment results show the top 10 BP terms, 10 CC terms and 10 MF terms. (B.) KEGG enrichment results show the 30 paths.
