# Supplementary figures and images for: Construction and validation of a metabolic risk model predicting prognosis of colon cancer
Source: Sci Rep. 2021 Mar 25;11:6837. doi: 10.1038/s41598-021-86286-z (PMC7994414; doi:10.1038/s41598-021-86286-z)

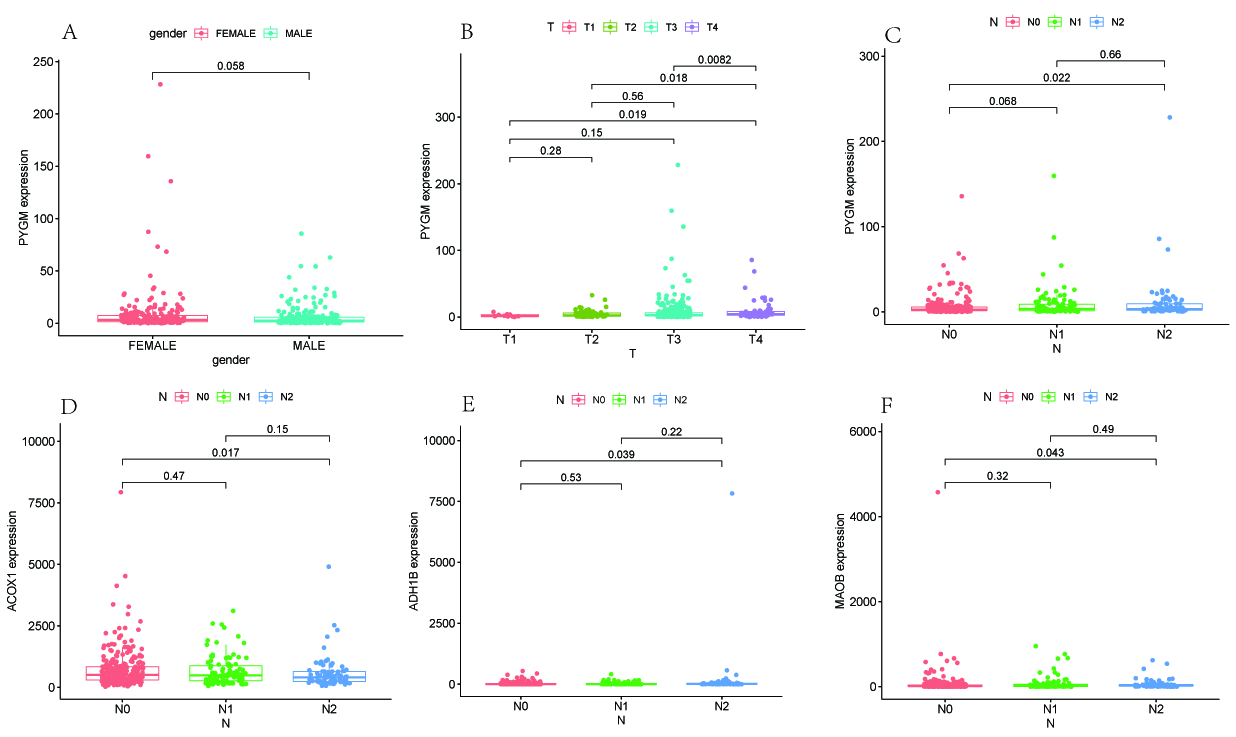

Supplement: Supplementary file 2 — Supplementary Figure 1. [file 41598_2021_86286_MOESM2_ESM.tif]

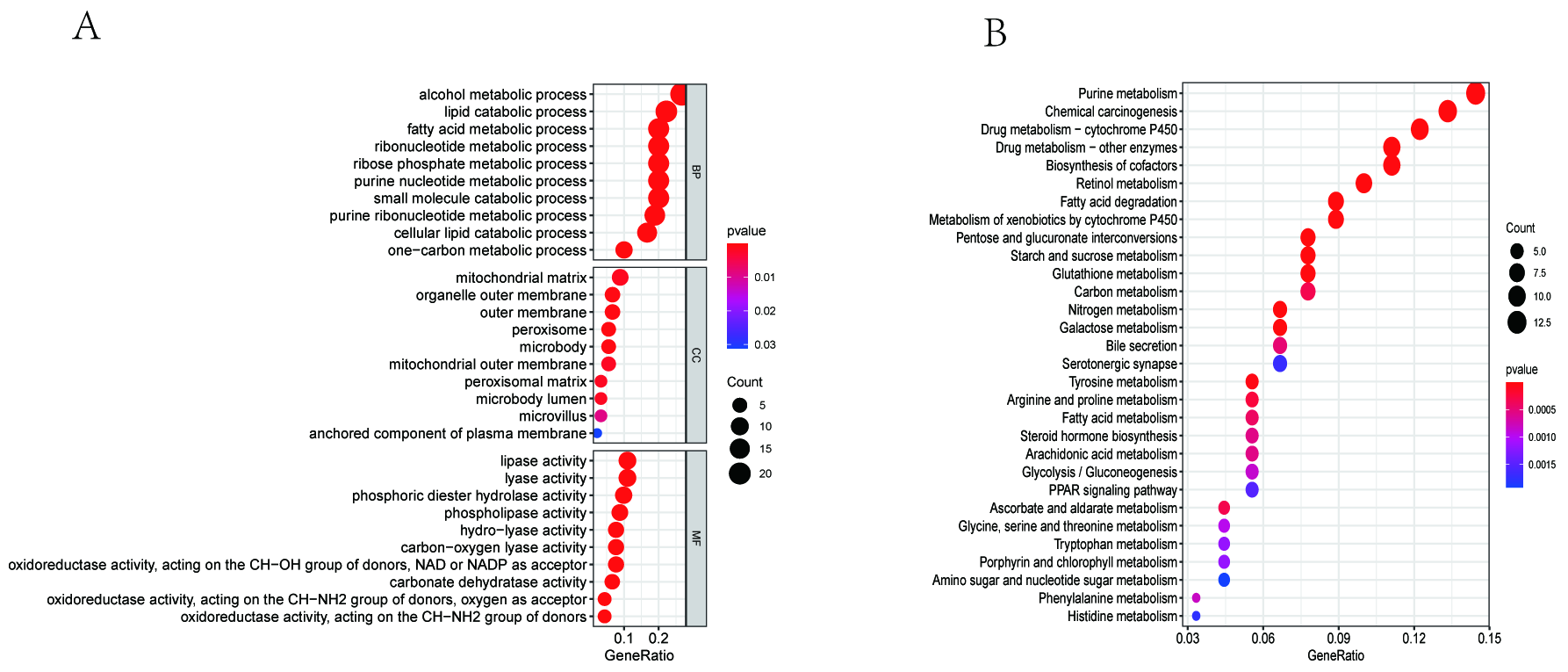

Supplement: Supplementary file 3 — Supplementary Figure 2. [file 41598_2021_86286_MOESM3_ESM.tif]
