# Supplementary material for: Construction and validation of a metabolic risk model predicting prognosis of colon cancer
Source: Sci Rep. 2021 Mar 25;11:6837. doi: 10.1038/s41598-021-86286-z (PMC7994414; doi:10.1038/s41598-021-86286-z)
Supplement: Supplementary file 4 — Supplementary Tables. [file 41598_2021_86286_MOESM4_ESM.docx]

Supplementary Table 1. The list of 753 metabolic genes from TCGA database

| DGKE | PYGL | GSTK1 | TXNDC12 | PLCG2 | GUCY2D |
| --- | --- | --- | --- | --- | --- |
| PRPS2 | IDO1 | TSTA3 | UGP2 | ALDH1A1 | LPCAT2 |
| ME2 | CYP1B1 | CRLS1 | GSTO2 | PLA2G3 | ENOPH1 |
| TXNRD1 | UPP1 | ACADSB | AK7 | CD38 | POLR2B |
| GAA | INPP1 | GALE | AKR1B1 | UCKL1 | CHKA |
| SAT2 | EHHADH | PTDSS2 | PGP | POLR2D | PLA2G4A |
| POLR1B | PCK1 | UROC1 | CYP4F3 | POLR2E | DLD |
| PPOX | AKR1A1 | PDE5A | PSAT1 | MTHFD1 | TRMT11 |
| HAGHL | PFKM | NME4 | ZNRD1 | GUSB | ACAA2 |
| ENTPD2 | CA7 | POLR2H | TBXAS1 | PNPT1 | NME3 |
| ADCY3 | MTHFD2L | CYB5R3 | MCEE | SEPHS1 | BPNT1 |
| PDE8A | AADAT | FECH | ALDOA | SMPD3 | POLR1E |
| SYNJ2 | HK3 | NNT | MTMR6 | MAT2A | NEU4 |
| GUK1 | UPP2 | DGUOK | ALOX12B | KYNU | PTGES |
| PDE6B | MDH1 | FMO5 | CYP2F1 | PFKFB4 | INMT |
| ADCY2 | CYP26A1 | GBE1 | MGST3 | PTGS2 | CDIPT |
| AOC3 | GNE | ADCY9 | ACSL5 | NEU1 | GSR |
| TYMS | POLD2 | MGST2 | MAOA | OAT | ADH5 |
| GPX2 | GALK2 | PNP | PDE6D | DAO | PIK3C2B |
| AMPD1 | SEPHS2 | ALDH3B1 | GPX7 | ME1 | CAT |
| NUDT12 | SMPD1 | GYS2 | SULT1A2 | MINPP1 | SUCLG1 |
| ACO2 | GLUL | ALDH3B2 | ACSL3 | NT5C2 | SGMS2 |
| POLA1 | MBOAT7 | DHRS9 | PLCG1 | DGKQ | POLR3H |
| PLA2G2A | METTL2B | GSTM3 | GLUD2 | GNPAT | CHST12 |
| SPTLC1 | PGD | POLR2G | AK2 | ALAS2 | PGM2 |
| EPHX2 | GLO1 | INPP4A | HEXB | ENTPD4 | LDHD |
| CYP2C18 | DGKD | PKM | CAD | PDE2A | NT5C |
| SAT1 | GAMT | ACOX3 | LPCAT1 | L2HGDH | LPCAT4 |
| CTPS2 | ASMT | ADH6 | UGCG | CERK | PAFAH1B2 |
| PLA2G2F | HK2 | AKR1B10 | PLCB1 | GPX1 | CBR3 |
| FADS2 | GDA | ADCY4 | DPYS | NAT2 | AMDHD1 |
| NAGK | ALDH4A1 | ACSL1 | LCMT2 | PFAS | B4GALT1 |
| PIP5K1C | TPO | CA14 | HIBCH | GPD1L | ACADS |
| ADH1C | LTC4S | GLA | CHPT1 | POLR3K | CNDP1 |
| DMGDH | PTGES2 | DTYMK | ENPP7 | GSTM5 | LPL |
| PGM2L1 | TPI1 | LAP3 | PAICS | ADCY7 | ENTPD5 |
| LIPG | ACHE | GPX3 | ARG2 | INPP5E | ALAS1 |
| BAAT | CHAT | PDE9A | CA9 | NADK | PFKL |
| PRDX6 | GPI | ACOT12 | ETNK1 | AMPD2 | HEXA |
| SGPL1 | AKR1C1 | MGLL | PRIM1 | ACACA | POLD3 |
| AHCYL1 | DCTD | ACADL | BDH2 | WARS2 | TK1 |
| CA5A | AOC1 | HADHB | UGT2B17 | TPH1 | ACPP |
| GGT6 | TREH | LDHAL6B | PIK3CB | PIP5K1A | ACSM3 |
| PIK3CG | NPR2 | CS | SULT1A1 | DEGS1 | UROS |
| MTAP | PLA2G2D | GPAT2 | TDO2 | PHOSPHO1 | GAL3ST1 |
| P4HA3 | GGCT | GCLM | APIP | ACADVL | FPGT |
| PAPSS1 | ACO1 | NEU3 | NMNAT2 | CYP2A6 | GPT |
| GANC | PIP4K2B | ETNK2 | NNMT | ACSM1 | DGAT2 |
| TH | AGPAT1 | PTEN | SCLY | POLR2C | PAFAH1B3 |
| ALDH3A2 | GSTP1 | POLR2I | GLUD1 | MAOB | ABAT |
| ACP5 | PLCE1 | PFKFB2 | OXCT1 | AGL | DDO |
| SMPD2 | CTH | PTGIS | POLR3C | PIKFYVE | PIPOX |
| PDE1A | FHIT | AACS | PLA2G15 | ISYNA1 | ASL |
| SHMT1 | ASPA | PNLIPRP2 | GCLC | GLS2 | PCYT2 |
| RDH16 | SPHK2 | PLA2G5 | POLE4 | ECI1 | ALDOC |
| POLR2A | AMD1 | GMDS | G6PC | IPPK | CYP26B1 |
| CYP2J2 | SRR | PPAT | SGPP2 | GUCY1A2 | INPPL1 |
| HPGDS | GSTZ1 | PHPT1 | ENPP1 | TRDMT1 | ME3 |
| ARSA | GNPDA1 | DHODH | NAT1 | GBA3 | LYPLA1 |
| RRM2 | RDH11 | AK3 | UXS1 | NMNAT1 | RDH8 |
| GNPNAT1 | PRIM2 | PDE10A | CYP2C9 | PC | RDH10 |
| GSTO1 | CEL | GMPS | DNMT3B | CHKB | DCK |
| GAD1 | ACSS3 | PNPLA3 | CA12 | LCAT | OGDHL |
| IMPDH1 | AGPAT2 | ACP6 | LCMT1 | BLVRA | ACSL6 |
| IMPA1 | PFKP | OPLAH | IMPDH2 | PIK3C3 | ENPP6 |
| RFK | AANAT | SPTLC2 | NIT2 | ADH1B | ACSM2A |
| ENTPD3 | HPD | OTC | CA4 | KMO | ALOX12 |
| POLR3GL | PIP5K1B | PCYT1A | RDH12 | HNMT | CBS |
| COMT | POLA2 | PCK2 | ADCY5 | NT5E | SUCLG2 |
| POLE2 | CYP2S1 | PDE8B | ALDH9A1 | BLVRB | PGS1 |
| PNPLA4 | AOC2 | AKR1C3 | CA5B | PIK3CD | POLR2L |
| ACSS2 | SGPP1 | UGT2A3 | ACSS1 | GPT2 | DGKA |
| ALDH6A1 | FMO2 | HCCS | XDH | GMPR | NOS1 |
| PLD2 | CPT1A | GGT1 | GOT1 | SPHK1 | PDE1C |
| ACSL4 | HMGCS1 | LPGAT1 | AKR1C4 | ATIC | PLCD3 |
| CPS1 | PAFAH2 | GSS | ALAD | ITPK1 | PFKFB3 |
| NME7 | HMGCS2 | ALDH1B1 | IL4I1 | CSAD | ENTPD1 |
| PLA2G6 | NAMPT | SI | ITPKB | TAT | GATM |
| TAZ | UGT1A1 | LPCAT3 | MLYCD | POLR3G | PDE3B |
| ADCY6 | UGDH | MBOAT1 | ITPA | CA2 | HEMK1 |
| LIPC | MTMR1 | ADK | GYS1 | HMOX2 | CPT1C |
| PDHB | POLR1D | PSPH | PGM1 | UMPS | HMGCL |
| ADPRM | ACACB | INPP5K | CYP2C8 | INPP5A | AHCY |
| DGKH | NANP | CYP2C19 | MIOX | PIP4K2C | MGST1 |
| POLR2J | PGM3 | PRPS1 | IPMK | NUDT9 | ADA |
| CKB | HDC | AGXT | GRHPR | CES2 | BST1 |
| HAGH | RENBP | FMO4 | DGKI | NME1 | RETSAT |
| BDH1 | COX10 | AOX1 | UROD | CDO1 | PLCB2 |
| PIK3C2G | ALDH5A1 | ACP2 | SDS | DUT | PLCB4 |
| ALDH2 | PDE6C | CKM | PLA2G12B | DHRS3 | PDE6G |
| PNLIPRP1 | ACAA1 | AMPD3 | POLR3B | CYP4F2 | AHCYL2 |
| NPL | MAT1A | RDH5 | NUDT2 | ENTPD6 | UCK1 |
| GPAM | PHGDH | MIF | POLR2F | GALK1 | GK |
| EPHX1 | FMO3 | POLE3 | ECHS1 | CHST13 | AMT |
| GCK | POLR3F | ACSM5 | PNMT | NMRK1 | HAAO |
| MGAM | ANPEP | TPMT | ACADM | POLR2K | PLCB3 |
| HAO2 | CPT2 | CYP3A4 | AGMAT | POLR1A | SHMT2 |
| ODC1 | G6PD | INPP5B | ACAT1 | APRT | ACYP1 |
| GMPR2 | PEMT | PAFAH1B1 | MTHFD1L | UGT2B15 | LDHB |
| ITPKA | ADSL | MDH2 | P4HA1 | SULT2B1 | PCYT1B |
| GART | DDC | IMPA2 | IDH2 | IDO2 | GNPDA2 |
| FBP1 | LCLAT1 | GSTA4 | ACP1 | GOT2 | PTGDS |
| CMPK2 | ACOX1 | PMM1 | DGAT1 | CP | AFMID |
| PTDSS1 | CA1 | ACY1 | AZIN2 | MAT2B | CYP2U1 |
| UGT1A6 | SRM | RRM1 | GALC | DNMT1 | IDH1 |
| PLD1 | B4GALT2 | ADO | B4GALT6 | ADI1 | PAPSS2 |
| ALDH18A1 | PDE7A | CMPK1 | NT5C3A | DLAT | DPYD |
| HMOX1 | DGKZ | SYNJ1 | CYP2D6 | CHDH | ECI2 |
| HK1 | PLCD4 | PTGS1 | NUDT5 | NANS | SMS |
| DNMT3A | GALT | FTH1 | MPST | GSTM1 | GNMT |
| CKMT2 | HGD | G6PC2 | PMM2 | LDHA | MTHFD2 |
| PDE7B | CDA | GPX4 | HMBS | HADHA | CBR1 |
| PDE4A | PI4KB | CYP2B6 | PDE6A | AK5 | SORD |
| MPI | FAH | PYCR1 | PI4KA | GLS | GUCY2C |
| PLA2G1B | NAGS | CYP2E1 | AGK | TXNRD2 | MTR |
| MTMR7 | GCAT | NOS3 | GCDH | TK2 | DGKG |
| CHIT1 | SUOX | UAP1 | ENPP2 | OGDH | ASNS |
| EARS2 | ALDH1A3 | POLD1 | NPR1 | METTL6 | ENPP3 |
| NMNAT3 | PLCD1 | CANT1 | GSTA1 | GSTM2 | POLR3A |
| HADH | FLAD1 | PDE1B | GGT7 | HYI | TYMP |
| ADH1A | INPP5J | PDE3A | KDSR | PIK3C2A | QPRT |
| PIP4K2A | MARS2 | AGXT2 | ACAT2 | POLD4 | AK1 |
| AGPAT3 | CDS1 | POLR1C | PDE4D | CMAS | GMPPA |
| POLE | NME6 | PLA2G7 | CPOX | POLR3D | PYGM |
| GSTM4 | CTPS1 | ACER3 | TYRP1 | GPD2 | HPRT1 |
| ASAH1 | MMAB | DEGS2 |  |  |  |
